# Genetic diversity and circulation of influenza A viruses in Italian pig farms: insights from surveillance and vaccination

**DOI:** 10.1186/s40813-026-00498-1

**Published:** 2026-03-13

**Authors:** Chiara Chiapponi, Alice Prosperi, Laura Soliani, Aurora De Mattia, Ada Mescoli, Camilla Torreggiani, Benedetta Cordioli, Ana Moreno, Luca Ferrari, Melania Andrani, Elena De Angelis, Valeria Cavalli, Giovanbattista Guadagnini, Davide Ponzoni, Emanuela Pileri, Matteo Ricchi, Paolo Borghetti, Paolo Martelli, Andrea Luppi

**Affiliations:** 1 Istituto Zooprofilattico Sperimentale della Lombardia e dell’Emilia Romagna (IZSLER), WOAH Reference Laboratory for Swine Influenza, Brescia, Italy; 2 Department of Veterinary Science, University of Parma, Parma, Italy; 3 Swine Practitioner, Vet Evolution, Brescia, Italy; 4 CEVA Animal Health, Milan, Italy; 5 Department of Chemistry, Life Sciences and Environmental Sustainability, University of Parma, Parma, Italy

**Keywords:** Swine, Influenza A virus, Longitudinal study, Subtyping, Genetic characterization, Immune responsiveness

## Abstract

**Background:**

The genetic diversity of influenza A viruses in swine (IAV-S) in Europe, including key subtypes such as H1N1, H3N2, H1N2, and H1N1pdm09 (which features H1 hemagglutinin genes from the H1A, H1B, and H1C genetic clades), presents challenges for vaccine development and raises concerns about potential swine-to-human transmission. IAV-S also affects the pork industry, requiring management practices such as vaccination and the adoption of biosecurity measures. To evaluate the dynamics of IAV-S infections in large pig herds and assess viral diversity and circulation patterns in pig farms, two Northern Italian farrow-to-finish farms with prior IAV-S infections participated in a 2022 longitudinal study. Sows and their piglets were sampled during farrowing and nursery. In 2023, a follow-up evaluated IAV-S circulation after implementing different vaccination strategies on the two farms, including vaccination of sows and piglets.

**Results:**

The circulation of multiple IAV-S strains into the two farms was observed, identifying H1BN2, H1AN1, H1CN1, and H1CN2, characterized by different genetic patterns. We confirmed that maternal immunity was ineffective in preventing virus circulation among piglets. HI tests showed variability in detecting strains, raising concerns about their specificity. After vaccination was implemented on both farms, we detected only H1CN2 strains, but with distinct genetic patterns.

**Conclusions:**

The study highlighted the genetic diversity of IAV-S strains circulating in Italian pig farms, including potential incursions from unknown external sources. Vaccination efforts resulted in broad antibody responses; however, as expected, they did not eliminate viral circulation. The diversity of lineage-derived H1C strains, along with the emergence of H1C2.4 strains, will require further investigation.

**Supplementary Information:**

The online version contains supplementary material available at 10.1186/s40813-026-00498-1.

## Background

The outbreak of the 2009 H1N1 influenza pandemic, which was traced back to swine, has highlighted the critical role that pigs play in the ecology and evolution of influenza A viruses (IAVs) [1]. Pigs are susceptible to infection by both avian and human influenza strains, serving as a ‘mixing vessel’ in which genetic reassortment can occur. This capacity of viral reassortment in swine has led to the generation of new influenza strains with pandemic potential [2].

The main subtypes of influenza A viruses in swine (IAV-S) circulating in European pig populations include:

- H1N1 (H1avN1 or H1CN1) [3]: this subtype has been endemic in European pig populations since the 1970s [4]. The 1C lineage, originating from an avian H1N1 virus, was first identified in Europe and subsequently spread to Asia. It became a dominant lineage in Eurasia (H1avN1 in Europe) and significantly influenced the current ecology of influenza A viruses. The strains 1C.1 and 1C.2 HA are typically paired with either the original avian-origin N1 NA or N2 NA, acquired from human seasonal viruses introduced in swine in the 1980s and 2000s. The 1C lineage demonstrates the role intercontinental spread and reassortment with avian and human viruses have in shaping the diversity of IAV-S [5].
- H3N2: similar to H1N1, H3N2 is also a significant subtype circulating in European pigs [4]. H3N2 viruses are characterized by genetic diversity and have been the subject of reassortment events, including those involving human-origin strains [3, 5, 6]. Swine H3N2 viruses are categorized based on the time of introduction of their ancestral human seasonal H3N2 virus; a human H3N2 lineage was established in European swine in the 1970s, later acquiring internal genes from the 1C H1avN1 swine virus. Most swine H3N2 viruses possess HA and NA genes derived from human seasonal H3N2 viruses, often from the same spillover event.
- H1N2 (H1huN2 or H1BN2): this subtype, often arising from reassortment events between different influenza lineages, has been reported in European pigs since the 1990s.

The 1B lineage of IAV-S originated from human seasonal H1N1 viruses in the UK in the 1990s (1B.1) and later in the US in the 2000s (1B.2) after these viruses crossed into swine populations. These viruses, distinct from classical swine lineages, have been reported in various European countries. 1B.2 HA clades in the US are paired with N2 NA genes from 1998/2002 human seasonal viruses. European 1B.1 HAs are mainly paired with a different human seasonal N2 NA from the 2000s. The 1B lineage highlights the ongoing transmission of influenza A viruses between humans and swine, leading to the emergence of new reassortant viruses with pandemic potential [4].

- H1N1pdm09 (H1AN1): the 1A lineage originated from the 1918 human Spanish flu pandemic. It established itself globally as the classical swine H1N1 (cH1N1) lineage. The 1A lineage includes the H1 HA of the 2009 pandemic H1N1 virus (H1N1pdm09). While a direct precursor was not found in pigs before 2009, viruses with similar gene segments were found in Mexican pigs between 2010 and 2014 [7, 8]. This subtype has continued to circulate and reassort with other swine influenza viruses, including in European pig populations. 1A HA clades are found with N1 NA genes (from classical swine or H1N1pdm09 lineages) or N2 NA genes (from 1998 or 2002 human seasonal lineages) [3, 9], adding complexity to IAV-S circulation in European pigs [4, 5].

The prevalence and distribution of all these subtypes can vary significantly across European regions and over time. Factors like husbandry practices, co-circulation of other respiratory pathogens, and vaccination strategies can influence the dynamics of IAV-S circulation [4].

This diversity poses significant challenges for swine health, vaccine development, and pandemic preparedness. Current human IAV vaccines often poorly match circulating IAV-S, raising concerns about their effectiveness in preventing swine-to-human transmission [3, 10]. Therefore, continuous surveillance of IAV-S, particularly in regions with high viral diversity and human-animal interaction, is crucial for monitoring viral evolution, assessing pandemic risk, and driving public health interventions.

Moreover, the circulation of IAV-S in swine farms has significant economic implications for the global pork industry. IAV-S infection can cause respiratory distress, fever, and reduced appetite in pigs, ultimately hindering growth. Infection can result in reduced productivity and performance, decreases in average daily weight gain, increases in antimicrobial usage for treating secondary bacterial infection and increased mortality rates, especially in young pigs [11].

Currently, strategies to mitigate the spread of IAV-S primarily rely on management practices. One of the main goals is to protect sows from infection and the consequences of influenza. Sow vaccination should be of dual benefit, reducing the risk of infection in piglets, thanks to both the direct protective effect of maternally derived antibodies and the indirect effects of reduced exposure pressure. However, the role of maternally derived antibodies (MDA) is complex: while they prevent clinical signs and may lower infection levels, they do not provide full protection against infection and can, in some instances, even increase viral shedding [12]. Consequently, the benefit of sow vaccination lies less in direct piglet immunity and more in reducing the overall infection pressure within the herd, thereby lowering the risk of piglet exposure. The infection dynamics in suckling and weaned piglets remain heavily dependent on management, specifically in preventing transmission from nurseries or older piglets [13].

In the framework of the ICRAD PIGIE project, we performed longitudinal monitoring for IAV-S circulation in two farrow-to-finish pig farms located in Northern Italy. The aim of the study was to evaluate the dynamics of IAV-S infections in large pig herds, in order to assess the viral diversity and its circulation patterns and the immunological response of pigs. The study was conducted in a first-round sampling in 2022, when neither farm performed IAV-S vaccination. In 2023, the farmers decided to implement a vaccination program, and an IAV-S virologic and serologic follow-up was performed. The effects of vaccination have been evaluated on the dynamics of infection, and not on clinical and performance parameters, which were not considered.

## Methods

### Description of the selected farms

Farm #1 was a farrow-to-finish herd with 350 sows, with one farrowing unit and one nursery unit. Farm #2 was a farrow-to-finish herd with 500 sows with 7 farrowing units and one nursery unit. Both farms were organized into a three-week batching system and had a fattening unit located on site.

In both farms, the farrowing and the nursery units were managed with an all-in/all-out flow, whereas in the fattening unit, there was a continuous flow. At the start of the study, none of the selected farms performed influenza vaccination. There was no reproductive failure or abortion, but practitioners reported respiratory symptoms in the nursery, sometimes severe, about 2 or 3 weeks after weaning, where viremic pigs for PRRSV were detected. The most critical stage was the nursery period, with a mortality as high as 8%. The farms registered a 2% mortality in the fattening unit, especially related to Hemorrhagic Bowel Syndrome and lameness. Fattening pigs were sold at 10 months of age at 175 kg/BW.

The sows were vaccinated with a PRRSV modified-live virus (MLV) vaccine every 3 months, and against parvovirosis, erysipelas and leptospirosis during lactation. Piglets were vaccinated against *Mycoplasma hyopneumoniae* and Circovirus porcine type 2 (PCV2) during lactation and at weaning, respectively. *Mycoplasma hyopneumoniae* vaccination was boosted at 50 kg/BW in the fattening unit with the same single-dose vaccine. The replacement gilts were produced on the farm, without introducing any pigs from external sources.

### Sampling and analyses

#### 2022 longitudinal sampling

The longitudinal study was performed under the Italian Ministry of Health Authorization No 315/2021-PR on two farms in Lombardy, Northern Italy, a region with the highest density of pig farms in Italy. The two farms were screened through M gene IAV real-time RT-PCR and virus sequencing in January 2022, confirming the circulation of influenza in the farrowing units and nursery pigs on both farms (Farm#1 H1B1.2.2N2; Farm#2 H1C2.4N2 and H1A3.3.2N1).

**Fig. 1:**
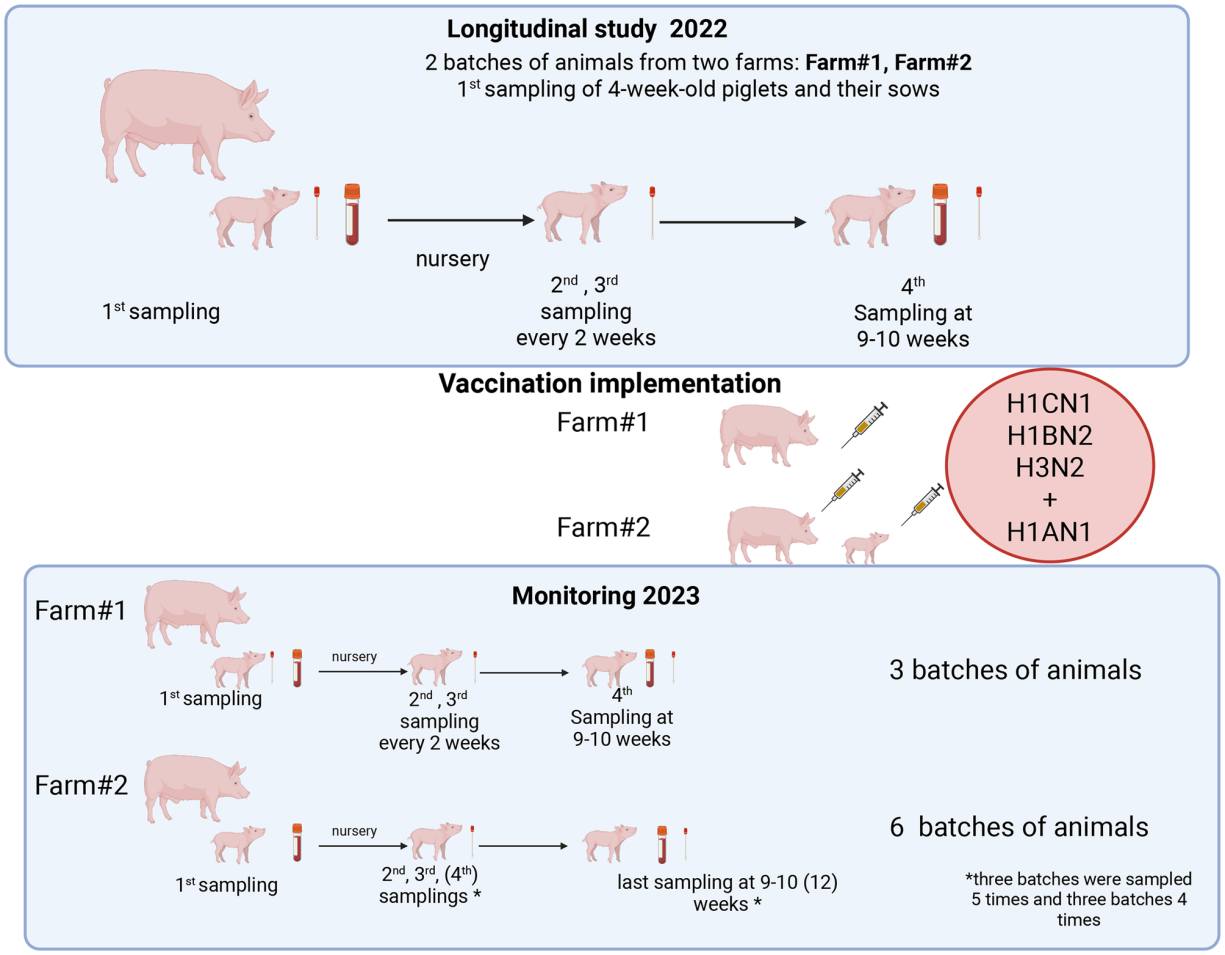
Visual representation of the sampling scheme adopted. Created in BioRender. Chiapponi, C. (2026) https://BioRender.com/obai4od

During the longitudinal study, two batches of piglets were sampled four times: once in the farrowing unit at 28 days of age, and three times, two weeks apart, in the nursery. Nasal swabs (SIGMA-Virocult® MW951SENT, Medical Wire & Equipment, 2025, UK) were collected at each sampling, while blood samples (collected in test tubes without anticoagulant) were taken during the first and last samplings. At the initial sampling, nasal swabs and blood samples were also collected from the respective sows (Fig. 1, Table 1). The swabs were kept refrigerated, while the blood samples were allowed to separate into serum and sent to the laboratory on the day of collection.

**Table 1:** Flow chart explaining the sampling scheme and laboratory tests. NP ELISA: Nucleoprotein ELISA; HI: hemagglutination inhibition; ELISPOT: enzyme-linked ImmunoSpot

Sampling_ID	Animal	Sample	TEST
Sampling 1	sows&piglets	blood	NP ELISA, HI (ELISPOT in Farm#2)
		nasal swabs	IAV Molecular screening, Virus isolation, sequencing
Sampling 2–3	piglets	nasal swabs	IAV Molecular screening, Virus isolation, sequencing
Sampling 4	piglets	blood	NP ELISA, HI (ELISPOT in Farm#2)
		nasal swabs	Molecular screening, Virus isolation, sequencing

Thirty-three animals per breeding group (3 piglets from 10 different litters, plus three more pigs from an eleventh litter, to cover mortality losses of animals during the study) were sampled on the two farms. Thirty samples were considered statistically necessary to determine the presence of swIAV on the farm, with a 95% confidence level and an estimated prevalence ≥ 10% within the farm. Sampling from the same farm across 2 different groups of animals was considered the minimum required to assess the evolution of viruses over time. The study of two farms was necessary to provide data on the circulation of at least two independent viral strains [14]. Whole blood was collected from selected batches in Farm #2, and peripheral blood mononuclear cells (PBMCs) were isolated and frozen on the same day of blood collection, as described below. According to the study protocol, animals exhibiting clinical signs compatible with PRDC would have been sampled additionally for diagnostic confirmation.

#### 2023 follow-up

The owners of the farms involved in the project, implemented IAV vaccination using two different approaches. Samples as described above were collected for diagnostic purposes and tested by the IZSLER laboratory to monitor influenza A virus circulation. Performance parameters were not available; we only collected information about the genetic diversity of virus circulation among the progeny. On Farm #2, it was possible to evaluate the viral diversity in the progeny when piglets were vaccinated (in addition to sows).

*Farm #1.* On the first farm, the sows were vaccinated against influenza. Sampling was performed to monitor viral circulation in the pre-weaning period and in the weaning/growing phases.

The sow and gilt vaccination schedule began with a first blanket vaccination using 2 ml intramuscular (IM) of Respiporc Flu 3 (CEVA Animal Health, Italy) and 1 ml IM of Respiporc FluPan (CEVA Animal Health, Italy), followed by a booster 3 weeks later. Once the basic vaccination was completed, a widespread booster vaccination was performed 4 months later with Respiporc Flu 3 (CEVA Animal Health, Italy) and then every 3 months with Respiporc FluPan (CEVA Animal Health, Italy) (twice by the end of the study).

Three consecutive batches of piglets were monitored from June 2023 to November 2023. The first batch was analyzed 3 weeks after the completion of the basic sow vaccination. For each group involved, a total of 33 piglets from 11 sows (3 piglets/sow) were sampled using nasal swabs at 28 days of age (S1 - before weaning), then at 6 (S2), 8 (S3), and 10 (S4) weeks of age. Blood samples were also taken from each pig in the first and fourth sampling. During S1, the 11 sows involved were sampled using blood and nasal swabs.

*Farm #2.* Both sows and piglets were vaccinated against influenza. Viral circulation during pre-weaning and weaning was subsequently monitored from June 2023 to January 2024.

**Fig. 2:**
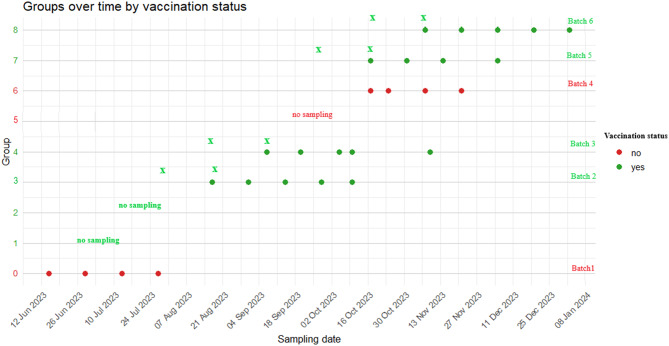
Vaccination and sampling scheme for pig batches of Farm #2. Sampling dates are represented by dots, vaccinated groups shown in green, and unvaccinated groups in red. X represents the vaccination date. Batch identification is on the right

The vaccination schedule with Respiporc Flu 3 (CEVA Animal Health, Italy) and Respiporc FluPan (CEVA Animal Health, Italy) of the sows and gilts was performed as described for Farm#1 (CEVA Animal Health, Italy). Vaccination of piglets started approximately 3 weeks after the basic vaccination of sows, with Respiporc Flu3 (CEVA Animal Health, Italy) and FluPan (CEVA Animal Health, Italy), to allow the development of adequate immunity in the breeding stock. Initially, 4 consecutive batches of piglets (groups = G1, G2, G3, and G4) were vaccinated with 2 ml of Respiporc Flu3 + 1 ml of Respiporc FluPan (CEVA Animal Health, Italy) + 1 ml of Respiporc FluPan (CEVA Animal Health, Italy). The next two consecutive batches of piglets (groups G5 and G6) did not receive any treatment, while the last two batches (groups G7 and G8) were vaccinated according to the same protocol used for groups from G1 to G4. For both vaccines, the first dose was administered around 7 days of age and the second around 28 days of age (the minimum time interval between the two vaccinations was 2 weeks) (Fig. 2).

Thirty-three piglets from 11 sows (3 piglets/sow) were identified for each group involved. These piglets were sampled by nasal swabs at 28 days of age (before weaning), then at 6, 8, and 10 weeks of age (groups G0, G7 and G8). In batch 2 (G3), batch 3 (G4), and batch 4 (G6), the pigs were also sampled at 12 weeks of age. Blood samples were also taken from each pig in the first and fourth sampling. At 12 weeks of age, in addition to nasal swabs, the sera was collected from each piglet (Figs. 1, 2).

At the time of the first sampling, the 11 sows involved were also sampled. From two selected batches in Farm #2, peripheral blood mononuclear cells (PBMCs), collected from 11 vaccinated sows, were isolated and frozen on the same day of blood collection, as described below.

### Virological study

After delivery to the laboratory, samples were stored at −70 °C until analysis. Viral RNA was extracted using a BioSprint 96 One-For-All Vet Kit (Indical Bioscience, Germany) according to the manufacturer’s instructions, and IAV-S M-gene real time RT-PCR was performed on RNA from the nasal swabs [15]. Positive samples were subtyped by multiplex RT-PCR [16] defining the lineage of HA (H1-1A, H1-1B, H1-1C) or H3 and the type of viral NA (N1 or N2). Virus isolation was attempted on SPF embryonated chicken eggs and suitable cell cultures (MDCK and CACO-2 cells) [17]. Amplicon sequencing was performed using an Illumina Miseq™ System (Illumina, Italy) from RNA of the original samples and isolated viruses, if available [16]. Viral genomes were amplified as previously described [18]. Genomic libraries were prepared using Nextera XT DNA Library Preparation Kit (Illumina, Italy), and sequencing runs were performed using MiSeq Reagent Nano Kit v2 (Illumina, Italy). For genetic analysis, the genome was divided into eight individual genetic segments for IAV-S. The lineage of each segment was determined by Blast analysis (https://blast.ncbi.nlm.nih.gov/Blast.cgi) against IAV-S influenza viruses present in GenBank as described previously, and the various IAV-S genotypes resulting from the different combinations of genes were identified by alphabetic or progressive order [5, 16, 19]. For lineage assignment, phylogenetic trees of the individual segments were inferred with the maximum likelihood (ML) method implemented in the IQ-TREE-2 v. 2.3.5 software [20, 21]. The trees were visualized using Figtree v1.4.4 (http://tree.bio.ed.ac.uk/software/figtree/) or CLC Genomic Workbench v.11 (QIAGEN, Italy). The origin of each segment was determined by its clustering with reference strains, and NA lineages were assigned as described [5] (N2g Ghent-like; N2s, Scotland-like; It-N2; A/swine/Italy/4675/2003; N1av, avian-like A/swine/Italy/311368/2013).

The virus gene sequences were aligned with the online version of MAFFT v.7 (https://mafft.cbrc.jp/alignment/server/index.html) [22, 23] with sequences in the IZSLER database since 2009 for swine influenza and with reference sequences in GenBank. The phylogenetic trees of the gene segments were obtained using the ML method in the IQ-TREE v2.3.5 software with bootstrap analysis of 1000 replicates [21].

The analysis of H1 viruses was also carried out online using the nomenclature described above at:https://www.bv-brc.org/app/SubspeciesClassification[24].

### Antigenic analysis

Selected H1 strains, isolated from the two farms in the longitudinal study performed in 2022:

- Farm #1 strain A/swine/Italy/91681-27/2022 H1N1 H1C2.5
- Farm #1 strain A/swine/Italy/109125-1/2022 H1N1 H1A3.3.2
- Farm #1 strain A/swine/Italy/75767-16/2022 H1N2 H1B1.2.2
- Farm #2 strain A/swine/Italy/49701-6/2022 H1N2 H1C2.4
- Farm #2 strain A/swine/Italy/49701-24/2022 H1N1 H1C2.1
- Farm #2 strain A/swine/Italy/29548-31/2022 H1N1 H1A3.3.2

were subject to HI testing, according to the WOAH manual protocol [25], using hyperimmune pig sera raised against A/swine/Italy/311368/2013 (H1N1 H1C2.1), A/swine/Italy/284922/2009 (H1N2, H1B1.2.2), A/swine/Italy/282866/2013 (H1N1 H1A3.3.2) and ferret sera against two recently isolated human strains: A/Ireland/87733/2019 and A/Norway/2685/2015 H1N1 (1A3.3.2) (kindly provided by Dr. Webb, St. Jude Children’s Research Hospital, Memphis through the CEIRR Influenza Reagent Distribution Program). Serum reactivity was considered low (HI titer = 20), moderate (HI titer from 40 to 80), or strong (HI titer ≥ 160) [26]. These hyperimmune sera exhibited HI titers against the homologous viruses ranging from 1:640 to 1:5,120.

### Serological testing - ELISA and hemagglutination inhibition (HI) test

The serum samples were tested using NP ELISA (ID-VET ID Screen^®^ Influenza A Nucleoprotein Swine Indirect), and titers were calculated according to the manufacturer’s instructions.

The hemagglutination inhibition (HI) test was performed using routine antigens (H1A3.3.2N1 A/swine/Italy/282866/2013, H1B1.2.2N2 A/swine/Italy/284922/2009, H1C2.1N1 A/swine/Italy/311368/2013, H3N2 A/swine/Italy/311349/2013) and using the antigens isolated from the farm (homologous antigens) (Table 2) with 0.5% turkey red blood cells [25, 28]. Samples were considered positive if the HI titer was equal or higher than 20. The threshold of HI titers ≥ 4-fold differences was considered to distinguish homologous titers from heterologous cross-reactions [29].

**Table 2:** Antigens used for serological testing for Farm#1 and Farm#2 in 2022 and 2023 samplings. HA clades were identified according to Anderson’s nomenclature [9] with the H1 subspecies classification (https://www.bv-brc.org/app/SubspeciesClassification) [24, 27]

Farm	Strain		Subtype	HA clade	Test
1	Routine	A/swine/Italy/311368/2013	H1N1	H1C2.1	HI 2022–2023
1	Routine	A/swine/Italy/284922/2009	H1N2	H1B1.2.2	HI 2022–2023
1	Routine	A/swine/Italy/282866/2013	H1N1	H1A3.3.2	HI 2022–2023
1	Routine	A/swine/Italy/311349/2013	H3N2	1970.1	HI 2022–2023
1	2022 Farm#1	A/swine/Italy/91681–27/2022	H1N1	H1C2.5	HI 2022, ELISpot 2022
1	2022 Farm#1	A/swine/Italy/109125-1/2022	H1N1	H1A3.3.2	HI 2022
1	2022 Farm#1	A/swine/Italy/75767–16/2022	H1N2	H1B1.2.2	HI 2022
1	2023 Farm#1	A/swine/Italy/213602-2/2023	H1N2	H1C2.4	HI 2023
1	2023 Farm#1	A/swine/Italy/296238-4/2023	H1N2	H1C2.4	HI 2023
2	Routine	A/swine/Italy/311368/2013	H1N1	H1C2.1	HI 2022–2023
2	Routine	A/swine/Italy/284922/2009	H1N2	H1B1.2.2	HI 2022–2023; ELISpot 2023
2	Routine	A/swine/Italy/282866/2013	H1N1	H1A3.3.2	HI 2022–2023
2	Routine	A/swine/Italy/311349/2013	H3N2	1970.1	HI 2022–2023, ELISpot 2022–2023
2	2022 Farm#2	A/swine/Italy/49701–6/2022	H1N2	H1C2.4	HI 2022, ELISpot 2022, VN
2	2022 Farm#2	A/swine/Italy/49701–24/2022	H1N1	H1C2.1	HI 2022, VN
2	2022 Farm#2	A/swine/Italy/29548–31/2022	H1N1	H1A3.3.2	HI 2022 ELISpot 2022, VN
2	2023 Farm#2	A/swine/Italy/299242-4/2023	H1N2	H1C2.4	HI 2023, ELISpot 2023
2	2023 Farm#2	A/swine/Italy/6097–4/2024	H1N2	H1C2.4	HI 2023, ELISpot 2023

### Serological testing - serum virus neutralization

Ten-fold dilutions of viruses were passed onto Madin–Darby canine kidney (MDCK) cells and viral titers were calculated according to the Reed and Muench method [28].

The serum virus neutralization (SN) assay was based on the ability of antibodies to inhibit the infection of MDCK cells by the influenza virus, as previously described. Briefly, 1:2 serial dilutions of inactivated swine serum samples were pre-incubated with a standardized amount of virus (100 TCID_50_) for 1 hour before adding MDCK cells (50,000 cells per well). The SN plates were examined under an inverted microscope every 24 hours. After 72 hours of incubation, a hemagglutination assay (HA) using turkey red blood cells was then performed on the supernatant of the SN plates to assess the presence of influenza A virus in infected MDCK cells and to evaluate the degree of neutralization by anti-influenza antibodies in the sera [28, 30].

### Serological testing - IAV-S-specific IFN-γ ELISpot assay upon* ex vivo *stimulation with swine influenza A viruses

The two batches of animals, sampled in Farm #2 in 2022, were tested by an IFN-γ ELISpot assay using the three strains isolated in the same farm (H1C2.4N2_2022, H1C2.1N1_2022, and H1N1 H1A3.3.2N1_2022) plus the reference-routine strain H3N2 A/swine/Italy/311349/2013 as control strain that was not detected during the study period (Table 2). The samples collected in 2023 from two batches of piglets, #1 and #2, sampled in Farm#2 at 4 weeks and 9–10 weeks of age, were tested using two (H1C2.4) recent strains isolated in this study, H1C2.4N2_2023_Gen35 and H1C2.4N2_2024_GenD, plus the reference strains H3N2 and H1B1.2.2N2 (Table 2).

Porcine PBMCs were isolated from 4 to 5 ml blood samples collected in lithium-heparin anticoagulant. Density gradient centrifugation was performed using Histopaque-1077^®^ (Merck, Italy) solution following the manufacturer’s protocol. Briefly, blood samples were layered onto an equal volume of Histopaque-1077^®^ and centrifuged at 400 x *g* for 30 minutes. The PBMCs were collected, washed with sterile PBS containing 1% fetal bovine serum (FBS), and resuspended in complete RPMI-1640 (cRPMI-1640) medium (Merck, Italy). The complete medium was composed of RPMI-1640 medium supplemented with 10% FBS, 2 mM L-glutamine, 100 μM non-essential amino acids, 50 μM 2β-mercaptoethanol, 100 U/ml penicillin G, 100 μg/ml streptomycin, and 0.25 μg/ml amphotericin B (Merck, Italy).

Isolated PBMCs were counted using an inverted optical microscope, and cell concentration was determined before downstream applications. Cell viability was consistently higher than 95%, as confirmed by Trypan blue exclusion. The PBMC samples were cryopreserved in a freezing medium using a Mr. Frosty^®^ device (Merck, Italy) and stored in liquid nitrogen for use.

The viruses used for cell stimulation in the ELISpot assay were quantified by digital PCR on a QIAcuity One instrument (QIAGEN). Briefly, RNA was extracted from 100 µl of cell culture supernatant as described above. Ten-fold dilutions (from 10^−1^ to 10^−4^) were performed in DNase/RNase-free water, and 5 µl of each dilution was tested using a 40 µl reaction with the QIAcuity^®^ One-Step Viral RT-PCR Kit (QIAGEN) using the same PCR primes (M + 25, *M*-124, *M*-124-SIV) and probe (M + 64) used for the diagnostic RT-PCR [15] at the final concentration of 0.4 µM, 0.2 µM, 0.2 µM, and 0.2 µM, respectively, in a 24-nanoplate well. The run was performed with the following thermal profile: 40 min at 50 °C and 2 min at 95 °C for 1 cycle, followed by 45 cycles at 95 °C for 15 sec and 60 °C for 30 sec with fluorescence acquisition. Viral concentration was calculated as copies/µl in the original sample. Before the ELISpot assay, viral suspensions were inactivated with 0.05% β-propiolactone (BPL) incubation for 24 h at 4 °C followed by incubation for 7 h at 37 °C [31]. The samples were stored at −80 °C until analysis.

To assess the activation of immune cells in response to influenza viruses, we measured the frequency of influenza A virus-specific IFN-γ secreting cells (SC) in PBMCs. This was achieved using an IFN-γ ELISpot assay based on previous protocols [32, 33] upon ex vivo stimulation with influenza virus isolates.

The PBMCs were plated at a density of 6 × 10^5^ cells/well (for piglets) or 4 × 10^5^ cells/well (for sows) in cRPMI-1640 (cRPMI-1640) supplemented with 10% FBS into 96-well plates (MultiScreen^®^ HTS-IP, Millipore, Italy) pre-coated overnight at 4 °C with 10 μg/ml anti-pig IFN-γ monoclonal antibody (clone P2G10, BD Pharmingen, Italy). The plates were then blocked with cRPMI-1640 for 2 hours at 37 °C. For antigen-specific recall, the cells were stimulated with influenza virus isolates at a multiplicity of infection (MOI) of 2 (for piglets) or 0.5 (for sows) in cRPMI for 40 hours at 37 °C in a 5% CO_2_ incubator. Following stimulation, the cells were discarded and the plates were washed with PBS + 0.05% Tween-20 (Merck, Italy) and incubated with 0.5 μg/ml biotinylated anti-pig IFN-γ monoclonal antibody (clone P2C11, BD Pharmingen) in PBS + 0.5% BSA for 1 hour at 37 °C. After washing, the plates were then incubated with a 1:1,500 alkaline phosphatase-conjugated streptavidin (cat. S921, Invitrogen-Thermofisher Scientific) in PBS + 0.5% BSA for 1 hour at 37 °C. Spot development was performed using a 1/100 BCIP/NBT substrate solution (BIO-RAD, Italy) for 7 minutes at room temperature in the dark, and the reaction was stopped with distilled water. The frequency of IFN-γ SC was determined using an AID^®^ ELISpot Reader and AID^®^ ELISpot Software v.6.0 (Autoimmun Diagnostika, Germany). Positive control wells contained PBMCs stimulated with 10 μg/ml phytohemagglutinin (PHA, Merck, Italy), while negative control wells contained unstimulated PBMCs. Background values from the negative control wells were subtracted from the stimulated wells. The immune response was expressed as the number of IAV-S-specific IFN-γ SC per 10^6^ PBMCs.

### Statistical analysis

Differences in the ELISpot data obtained in PBMCs stimulated with each different virus strains were determined at the two different time points using the SPSS software (IBM, v.27) and the Friedman test, followed by post hoc analysis using the Dunn test for pairwise comparisons incorporating Bonferroni’s correction. The Wilcoxon sum rank test was instead used for each virus in order to determine if the differences in ELISpot data among the virus strains at 4 and 8–10 weeks were statistically significant. *p*-values < 0.05 were considered statistically significant [34, 35]. Animals that died during the study, and sampled only once, were excluded from the statistical analysis. As the expected antibody response to the different strains was unknown, and to capture the biological diversity among the tested strains and piglets while minimizing the use of animals, a convenience sample of 30 piglets per group was chosen.

## Results

Some animals, sampled during the study, died (mortality ranged between 3 and 36%, see Supplementary file S1- farms’ positivity sheets) with enteric/septicemic clinical signs not attributable to PRDC.

### Virological longitudinal study − 2022

**Fig. 3:**
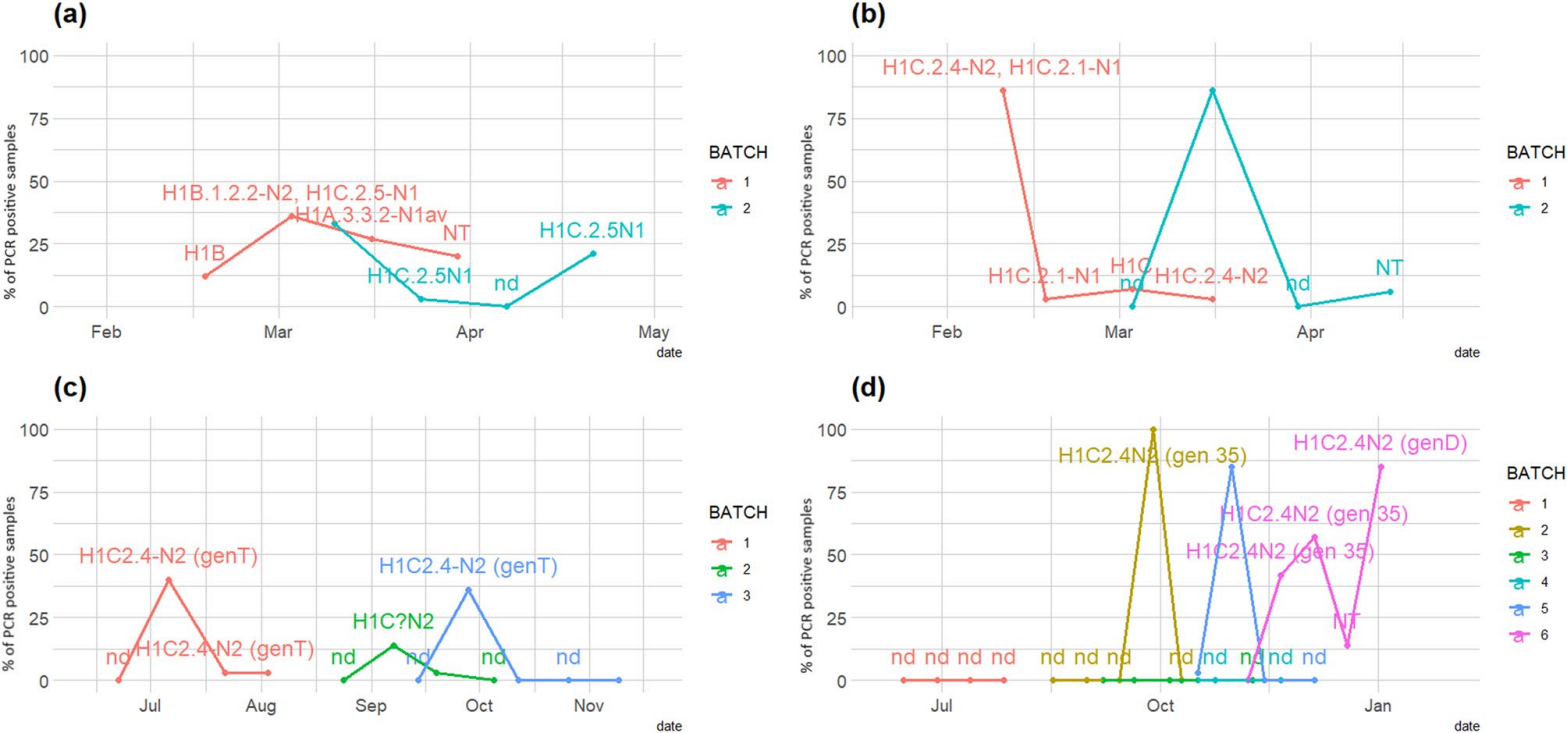
Percentage of PCR-positive samples detected in each analyzed batch in 2022 in Farm#1 (**a**) and in Farm#2 (**b**) and in 2023 in Farm#1 (**c**) and in Farm#2 (**d**). The detected viral subtypes are reported for each sampling (detected positivity, NT: non-typeable, nd: not detected)

Farm#1: Multiple H1 lineages of IAV-S were detected in the farm during the consecutive samplings (Fig. 3a): after initial circulation of H1B1.2.2N2 strains (present also in the January 2022 screening), H1C2.5N1 and H1A3.3.2N1 were detected in Batch 1. In Batch 2, only H1C2.5N1 was detected. The influenza PCR prevalence in the four samplings was 12, 36, 27, and 20% in Batch 1 and 33, 3, 3, and 21% in Batch 2, respectively.

Farm#2: After detection of H1C2.4N2 and H1A3.3.2N1 during the initial January 2022 screening, in the longitudinal sampling, H1C2.1N1 and H1C2.4N2 were detected, sometimes simultaneously (Fig. 3b). Mixed infections were observed. The influenza PCR positivity in the four samplings was 83, 9, 7, and 3% in Batch 1 and 0, 86, 0, and 6% in Batch 2, respectively.

Persistently infected animals were not detected at any sampling time.

**Fig. 4:**
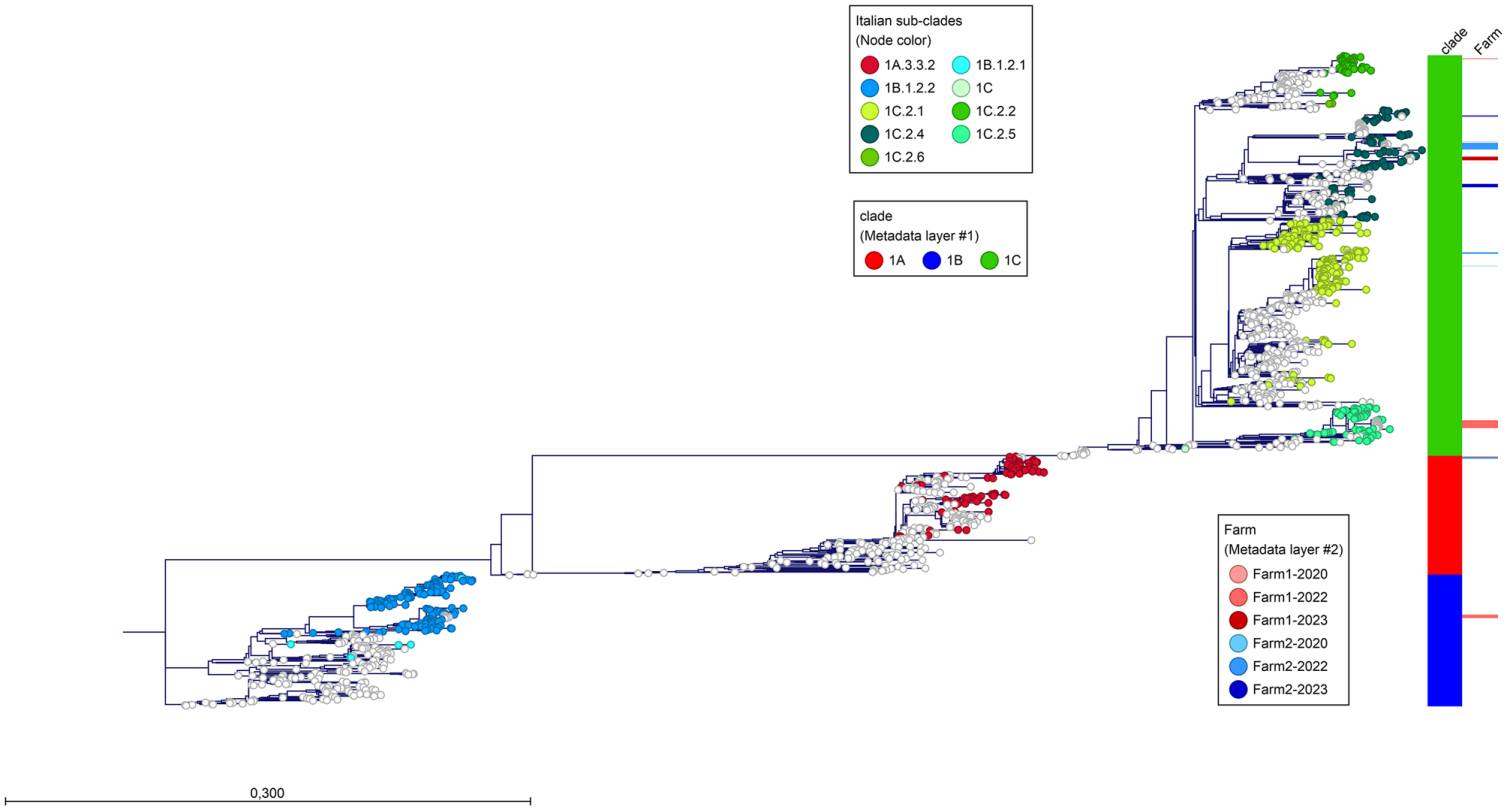
ML phylogenetic tree of 1738 HA-H1 sequences (1,518 nucleotides) for strains analyzed in this study (783 Italian strains and 83 sequences obtained in this study) with the identification of the H1 sub-clades of the strains collected in Farm#1 and Farm#2. The tree was inferred with European reference sequences retrieved from GenBank collected in the years 2009–2024 with the reference sequences used to construct the global swine H1 clade classification reference tree, which is used to classify the clades of the HA-H1 viruses. Italian sequences from 2004 to 2009 are represented by colored dots and sequences retrieved from GenBank are shown as white dots. HA clades are represented by colored bars: 1A red, 1B blue, 1C green, and the Italian HA1–H1 sub-clades are represented by different dot colors. Sequences from the Italian farms of the study are represented by red bars for Farm#1 (in three tones: light red is for the virus isolated in 2020, red for samples from 2022, and dark red for samples from the 2023–2024 follow-up), blue bars for Farm#2 (in three tones: light blue is for the virus isolated in 2020, blue for samples from 2022 and dark blue for samples from the 2023 follow-up)

Whole genome sequencing of the detected strains was performed on 83 samples, 44 from Farm#1 and 39 from Farm#2. The HA clade assignment and the phylogenetic analysis allowed us to identify the multiple genetic H1 clades circulating in the study period (Fig. 4). Moreover, the NA and internal gene origins were summarized to provide a picture of the genotypic variants circulating (Table 3).

**Table 3:** Genotypic combinations of the genes HA, NA, PB2, PB1, PA, NP, MP, and NS were detected in this study in the farms (F1 and F2) in the study of 2022 and in the follow-up in 2023–2024. HA sub-clade definition has been defined in https://www.bv-brc.org/ as described [9, 29]. NA lineages were assigned as described [5] (N2g ghent-like; N2s, Scotland-like; it-N2; A/swine/Italy/4675/2003; N1av, avian-like A/swine/Italy/311368/2013). The alphabetic genotype nomenclature has been added as previously described with letters and numbers [16, 19, 36]

Year	Farm	Genotype								
		Nomenclature	HA	NA	PB2	PB1	PA	NP	MP	NS
2022	F1	F	1B1.2.2	It-N2	av	av	av	av	av	av
		S	1A3.3.2	av	pdm	pdm	pdm	pdm	pdm	pdm
		A	1C2.5	av	av	av	av	av	av	av
	F2	S	1A3.3.2	av	pdm	pdm	pdm	pdm	pdm	pdm
		D	1C2.4	N2g	av	av	av	av	av	av
		A	1C2.1	av	av	av	av	av	av	av
2023–2024	F1	D	1C2.2	N2g	av	av	av	av	av	av
		T	1C2.4	N2g	pdm	pdm	pdm	pdm	pdm	pdm
	F2	35	1C2.4	N2g	pdm	av	pdm	pdm	pdm	pdm
		D	1C2.4	N2g	av	av	av	av	av	av

**Fig. 5:**
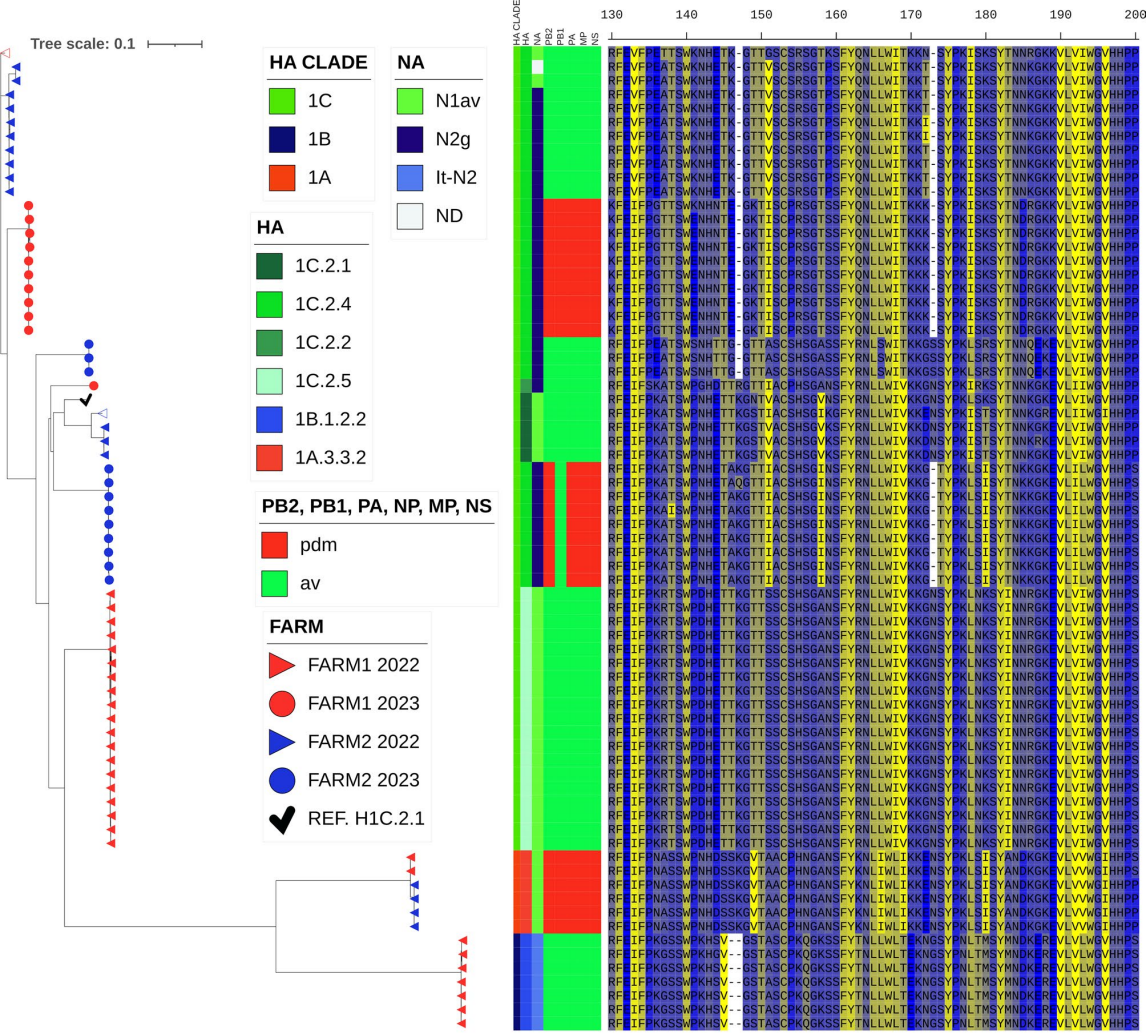
ML phylogenetic tree of 83 HA-H1 sequences (1,518 nucleotides) for strains from Farms#1 and #2 characterized in this study (2022 and 2023). The tree was inferred with the Italian reference strain H1C2.1N1 A/swine/Italy/311368/2013 H1N1 (✓). HA clades are represented by colored bars: 1A orange, 1B blue, 1C green, and different sub-clade colors are in legend blocks. Sequences from the Italian farms of the study are represented by a red triangle for 2022 samples Farm#1, a red circle for 2023 Farm#1, a blue triangle for 2022 Farm#2, and a blue circle for 2023 Farm#2. The genotype pattern is represented by colored blocks. HA sub-clade definition has been defined in https://www.bv-brc.org/ as described [9, 29]. NA lineages were assigned as described [5] (N2g ghent-like; N2s, Scotland-like; it-N2; A/swine/Italy/4675/2003; N1av, avian-like A/swine/Italy/311368/2013). The protein alignment (130–200 aa) is shown on the right-hand side of the diagram, along with the different putative aa deletions of the HA sub-clades (H1C2.4, $\Delta$147, $\Delta$173, and H1B1.2.2, $\Delta$146–147) (-)

The phylogenetic analysis of the HA genes (Fig. 4) showed that the strains obtained from the study belonged to different contemporary clades that are known to be circulating in Italy [16]. The combination of internal genes revealed that the strains exhibited different reassortment patterns, with 6 genotypes detected (Fig. 5). The H1A3.3.2N1 strains, detected in this study in both farms, belonged to the recently described reassortant strain detected in Italy and Denmark [16, 37] characterized by HA, PB2, PB1, PA, NP, MP, NS genes of H1N1pdm09 origin and NA gene of avian-like origin. H1B1.2.2N2 was the endemic H1N2 strain detected only in Italy, circulating since 2003 and characterized by a double aa deletion in the HA1 at the positions 146 and 147 ($\Delta$146–147) [5, 38].

The H1C strains showed multiple genetic patterns; H1C2.1 and H1C2.5 were found in combination with N1 and internal genes of avian-like origin. H1C2.2 and H1C2.4 were reassortant strains with N2 genes and avian-like or H1N1pdm09 internal gene combination (H1C2.4).

The H1C2.4 strains detected in 2022 were characterized by the same aa deletion pattern ∆147, ∆173 in the HA1 region (Fig. 5). The deletion ∆147 was close to the previously described antigenic site Sa and Ca2 of the HA1 gene (Table 4), and ∆173 was within the Sa putative antigenic site of the H1 protein [29].

**Table 4:** Detailed translation of the antigenic sites of HA1 [39] genes of the H1C lineages detected in the study. The routine strain A-swine-Italy-311368-2013-H1N1-1C2.1 was considered as reference and mutations and deletions are highlighted in bold. The ∆173 deletion of H1C2.4 strains is in the Sa antigenic site, and ∆147 deletion is close to Sa and Ca2

	Cb (87–88)		Ca2 (154–159)						Sa															Sb (201–212)												Ca1 (183–254)													Ca2 (238–239)	
									(141–142)		170–174)						(176–181)																																	
A-swine-Italy-311368-2013-H1N1-1C2.1	L	L	S	H	S	G	V	N	P	N	K	K	**G**	N	S	P		K	L	S	K	S	T		D	S	D	Q	Q	T	L	Y	Q	N	N	T	N	N	K	G	S	S	K	Y	D	Q	G	R		E
F1-2020-H1N1-1C2.4	L	L	S	**R**	S	G	**T**	**K**	**K**	N	K	K	**N**	-	S	P		K	**I**	S	K	S	T		**Y**	**N**	D	Q	Q	**A**	L	Y	Q	**S**	N	T	N	N	**R**	G	**T**	S	K	Y	D	Q	G	R		**N**
F1-2022-H1N1-1C2.5	L	L	S	H	S	G	**A**	N	P	**D**	K	K	G	N	S	P		K	L	**N**	K	S	**N**		D	**N**	D	Q	Q	**A**	L	Y	Q	N	N	**I**	N	N	**R**	G	S	S	K	Y	D	Q	G	R		**N**
F1-2023-H1N2-1C2.2	L	L	**P**	H	S	G	**A**	N	P	**G**	K	K	G	N	S	P		K	**I**	**R**	K	S	T		**E**	S	D	Q	Q	T	L	Y	Q	N	N	T	N	N	K	G	S	S	K	Y	**S**	Q	G	R		**N**
F1-2023-H1N2-1C2.4	L	L	**P**	**R**	S	G	**T**	**S**	**K**	N	K	K	**K**	-	S	P		K	**I**	S	K	S	T		**Y**	**N**	D	Q	Q	T	L	Y	Q	**S**	N	T	N	**D**	**R**	G	**T**	S	K	Y	D	Q	G	R		**N**
F2-2022-H1N1-1C2.4	L	L	S	**R**	S	G	**T**	**P**	**K**	N	K	K	**T**	-	S	P		K	**I**	S	K	S	T		**Y**	S	D	Q	Q	**A**	L	Y	Q	**S**	N	T	N	N	K	G	**T**	S	K	Y	D	Q	G	R		**N**
F2-2023-H1N2-1C2.4-GEN35	L	L	S	H	S	G	**I**	N	P	N	K	K	G	-	**T**	P		K	L	S	**I**	S	**N**		**Y**	**R**	D	Q	**E**	**A**	L	Y	Q	N	N	T	N	K	K	G	S	**P**	K	Y	D	Q	G	R		**N**
F2-2023-H1N2-1C2.4-GEND	L	L	S	H	S	G	**A**	**S**	**S**	N	K	K	G	S	S	P		K	L	S	**R**	S	T		D	**R**	D	Q	L	T	L	Y	Q	N	**D**	T	N	N	**Q**	**E**	S	S	**T**	Y	D	Q	G	R		**D**

### Virological follow-up study − 2023

The virologic status was slightly different after vaccination implementation in both farms. All the strains detected in both farms were H1CN2 viruses characterized by distinct gene combinations. H1A or H1B strains were not detected in the follow-up period.

In Farm#1 (Fig. 3c), where sow vaccination was implemented, we detected IAV-S circulation among piglets in all the sampled batches. From July to November 2023, two distinct H1CN2 strains circulated. To help viral differentiation, we adopted the previous genotypic nomenclature [5, 16, 36].

The first detected strain was H1C2.4N2 (Genotype T) (Table 3), detected in Batches 1 and 3. This was followed by the circulation of H1C2.2N2 (genotype D) in the second sampled batch.

On Farm#2, 3 out of 6 sampled batches of piglets showed IAV-S circulation. In particular, Batches 2, 5, and 6 were positive by PCR (Fig. 3d). The strain circulating for a long period (September-December 2023) was an H1C2.4N2 (genotype 35), which infected the piglets of the different groups consecutively in three waves of viral circulation in Batches 2, 5 and 6. In January 2024, during the last sampling in Batch 6, we detected a high proportion (85%) of animals positive for a different subtype, H1C2.4N2 (genotype D).

The HA phylogenetic analysis showed that the strains detected in the two farms belonged to different subclades. Despite being assigned to the same 1C2.4 subclades, the viruses circulating on the farms had different origins (Figs. 4–5). During the follow-up period after vaccine implementation, 4 strains were detected circulating among the sampled piglets (2 per farm). The HA H1C2.4 detected in Farm#1 possessed both the deletions ∆147 and ∆173, while in Farm#2, the two H1C2.4 detected, genotype 35 and genotype D, had single deletions ∆173 and ∆147, respectively (Fig. 5). The ∆173 deletion was into the previously described antigenic site Sa of the HA1 gene [39] (Table 4) and ∆147 was in a region between the Sa and Ca2 antigenic sites.

The H1C2.2 strain detected in Farm#1 had no aa deletion (Fig. 5). Interestingly, genotype 35 circulating in Farm#2 showed a reassortant pattern with internal genes of HA-1C, N2, and pandemic origin, except for the PB1 gene of avian-like origin. The HA protein had no fixed mutation from Batch 2 to Batch 6.

### Antigenic analysis of selected IAV-S isolates

Four out of five isolated from the two farms in the longitudinal study performed in 2022 reacted in the HI test with at least one of the two swine hyperimmune sera, with titers ranging from 40 to 640. The Farm#2 strain A/swine/Italy/49701–6/2022 (H1C2.4N2) reacted with a moderate titer with the 1C2.1 antiserum. The Farm#2 A/swine/Italy/29548–31/2022 strain (1A3.3.2N1) was the only strain that didn’t react. None of the tested isolates reacted with the hyperimmune sera against the human pandemic-origin strains (Table 5). The only significant reactivity with these hyperimmune sera, obtained with 1A3.3.2 human pandemic strains, was observed by testing the routine antigen A/swine/Italy/282866/2013 (H1A3.3.2).

**Table 5:** HI titers of selected strains (Farm#1 strain A/swine/Italy/91681–27/2022 H1N1 H1C2.5, Farm#1 strain A/swine/Italy/109125-1/2022 H1N1 H1A3.3.2, Farm#1 strain A/swine/Italy/75767–16/2022 H1N2 H1B1.2.2, Farm#2 strain A/swine/Italy/49701–6/2022 H1N2 H1C2.4, Farm#2 strain A/swine/Italy/49701–24/2022 H1N1 H1C2.1, Farm#2 strain A/swine/Italy/29548–31/2022 H1N1 H1A3.3.2) against the hyperimmune swine strains: A/swine/Italy/282866/2013 (1A3.3.2), A/swine/Italy/284922/2009 (1B.1.2.2), A/swine/Italy/311368/2013 (1C.2.1), and against two recently isolated human strains: A/Ireland/87733/2019 and A/Norway/2685/2015 H1N1 (1A3.3.2). In addition, the routine antigens A/swine/Italy/284922/2009 H1N2 (1B1.2.2), A/swine/Italy/311368/2013 H1N1 (1C2.1), A/swine/Italy/282866/2013 H1N1 (1A3.3.2) were used. These hyperimmune sera exhibited HI titers against the homologous viruses that ranged between 1∶640 and 1∶2560 (in bold)

		SERUM	A/swine/Italy/282866/2013	A/swine/Italy/284922/2009	A/swine/Italy/311368/2013	A/Ireland/87733/2019	A/Norway/2685/2015
		**HA clade**	**1A3.3.2**	**1B1.2.2**	**1C2.1**	**1A3.3.2**	**1A3.3.2**
**Virus**	**Subtype**		**H1N1**	**H1N2**	**H1N1**	**H1N1** **Human seasonal strain**	**H1N1** **Human seasonal strain**
**Routine** **A/swine/Italy/282866/2013**	H1pdmN1pdm	1A3.3.2	**5120**	NEG	160	640	320
**Farm#2** **A/swine/Italy/29548–31/2022**	H1pdmN1	1A3.3.2	NEG	NEG	NEG	NEG	NEG
**Routine** **A/swine/Italy/284922/2009**	H1huN2	1B1.2.2	NEG	**640**	NEG	ND	ND
**Farm#1** **A/swine/Italy/75767–16/2022**	H1huN2	1B1.2.2	20	640	NEG	NEG	NEG
**Routine** **A/swine/Italy/311368/2013**	H1avN1av	1C2.1	80	NEG	**1280**	80	NEG
**Farm#1** **A/swine/Italy/91681–27/2022**	H1avN1	1C2.5	40	NEG	320	40	NEG
**Farm#2** **A/swine/Italy/49701–24/2022**	H1avN1	1C2.1	320	NEG	1280	NEG	NEG
**Farm#2** **A/swine/Italy/49701–6/2022**	H1avN2	1C2.4	NEG	NEG	40	NEG	NEG

### Serological analysis − 2022

Farm#1. In Batch 1, 100% of the sows showed antibodies against IAV, while 91 and 94% of the piglets were ELISA-positive in Sampling 1 and 4, respectively. In Batch 2, 100% of sows and piglets were positive in sampling 1 (4 weeks of age), while 70% were positive in sampling 4 at 9–10 weeks, and the average ELISA titers decreased.

**Fig. 6:**
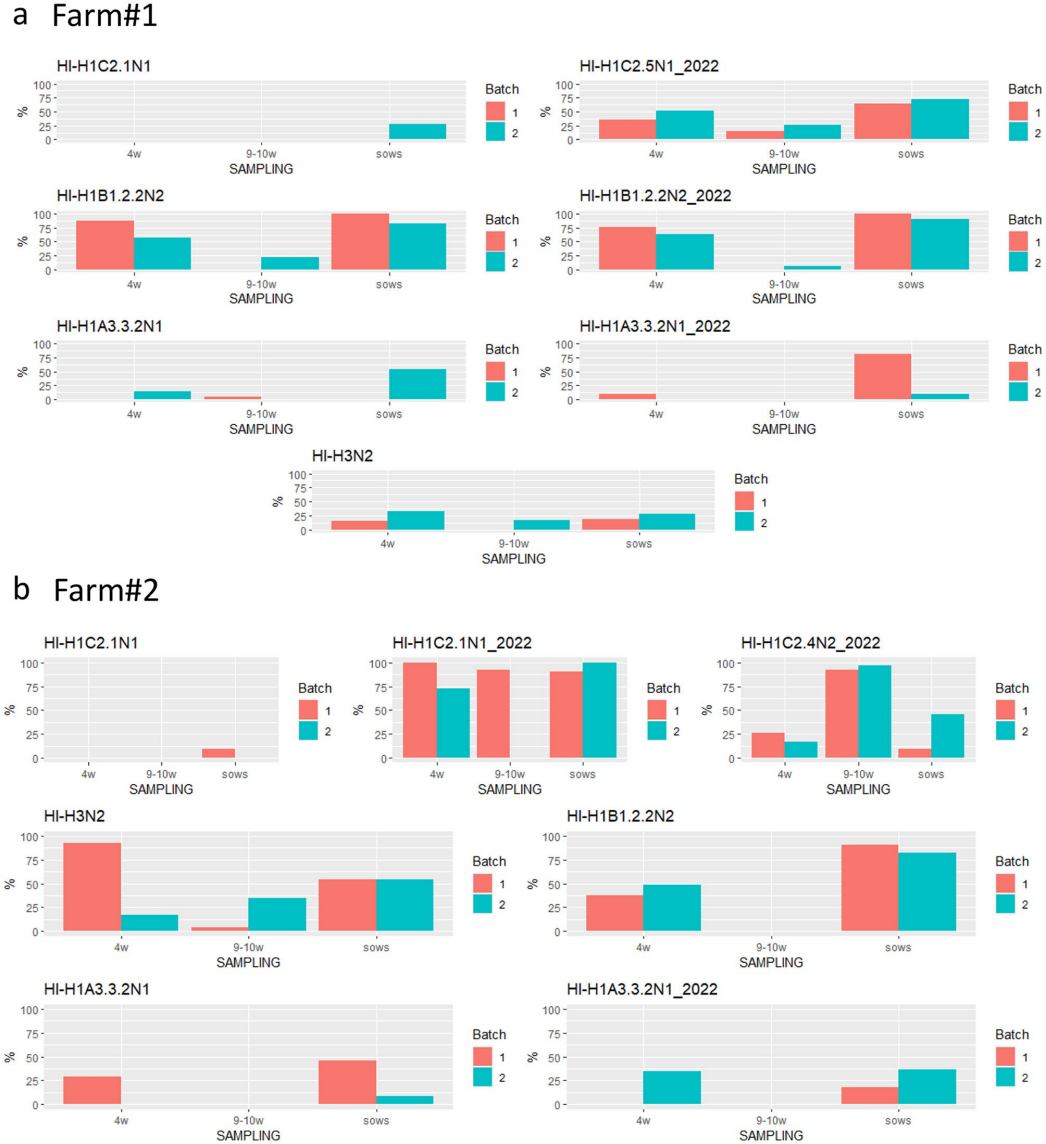
Percentage of positive animals (HI titers ≥ 20) per batch per sampling among piglets (4 weeks, 9–10 weeks) and sows obtained using different viral antigens in the test in Farm#1–2022 (**a**) and in Farm#2–2022 (**b**)

The HI was performed using seven antigens, four routine antigens and three homologous antigens isolated in the study period in the Farm#1. The HI analyses showed different rates of piglet positivity to all four subtypes in the Sampling 1 (Fig. 6a). The HI titers from the samples collected in the last sampling from the piglets showed no signs of seroconversion (data not shown). The use of a homologous strain H1C2.5N1 2022 antigenic variant isolated in this farm, showed HI titers in both piglets and sows at the first collection, while negative results were obtained using the routine strain H1C2.1N1 (Fig. 6a).

Farm#2. In Batch 1, the ELISA test showed 100% of sows, 93 and 100% of piglets serologically positive in Sampling 1 (4 weeks of age) and 4 (9–10 weeks of age), respectively. In Batch 2, 100% of piglets and sows were serologically positive in Sampling 1, while 69% resulted positive in the last sampling with low titers.

The HI test using the H1C2.1N1 2022 isolated in Farm#2, showed a large number of positive animals among sows and piglets (Fig. 6b). Moreover, seroconversion to the H1C2.4N2 2022 strain isolated in the farm, which was slightly recognized by maternal immunity in the first sampling, was demonstrated in both groups of sampled piglets. In detail, 81% of Batch 1 and 93% of Batch 2 animals had an increased HI titer ≥ 4 (data not shown).

To confirm the HI data, a VN test was performed on the three homologous viruses isolated in Farm#2. The test confirmed that 70 and 63% of the Batch 1 and Batch 2 animals, respectively, seroconverted against the H1C2.4N2 strain, with at least a 4-fold increase in titer.

### Serological analysis 2023

**Fig. 7:**
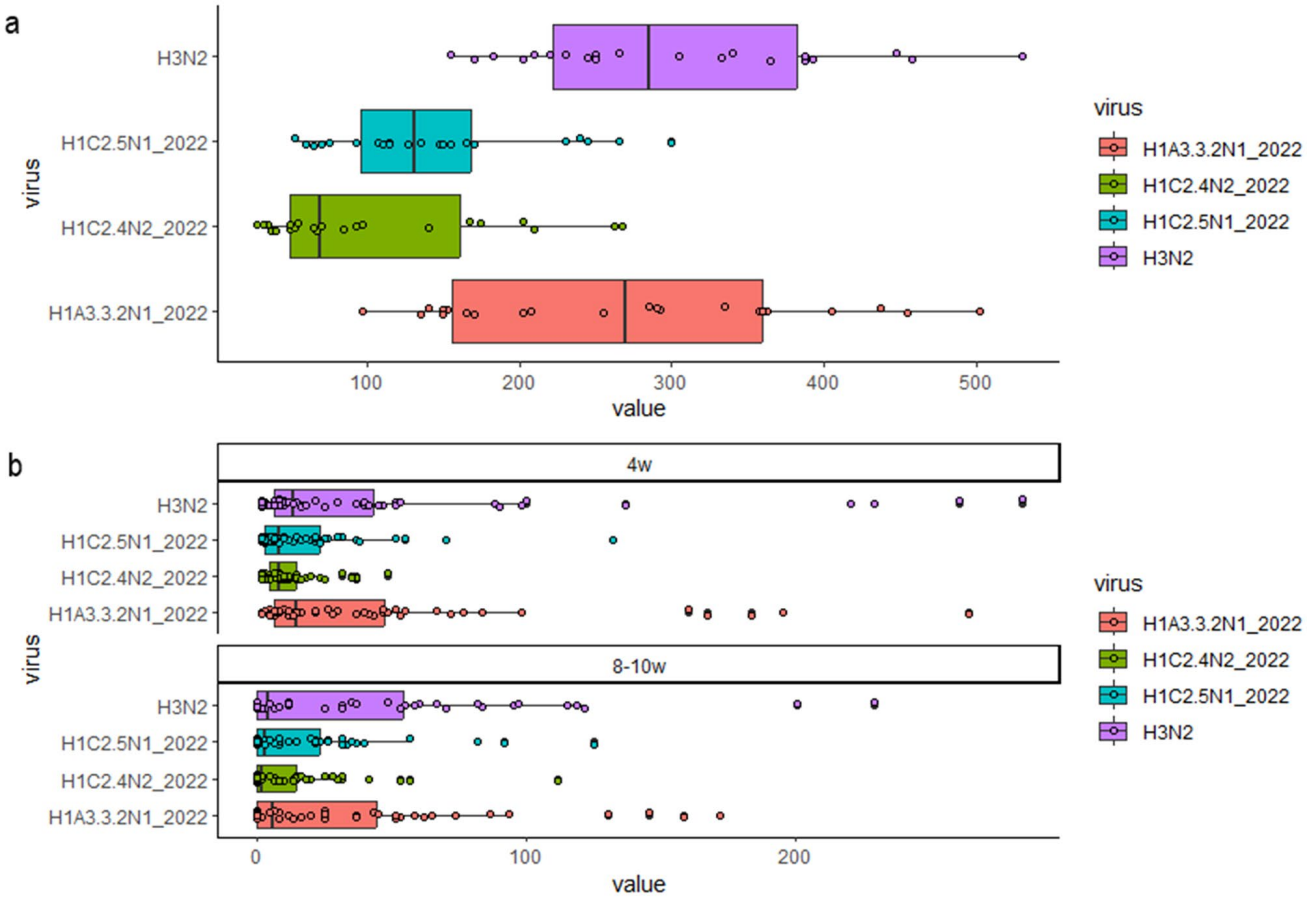
**a**) Sow ELISpot results. IFN-γ secreting cells/10^6^ PBMC for the two batches of Farm#2–2022 sows. Cells were stimulated with 0.5 MOI of four viruses. **b**) Piglet ELISpot results. IFN-γ secreting cells/10^6^ PBMC for the two batches of Farm#2 piglets. Cells were stimulated with 2 MOI of 4 viruses: H1A3.3.2N1 2022 A/swine/Italy/29548–31/2022, Routine H3N2 A/swine/Italy/311349/2013, H1C2.4N2 2022 A/swine/Italy/49701–6/2022, H1C2.1N1 2022 A/swine/Italy/49701–24/2022

In the samples collected on Farm#1, where sow vaccination was performed, the sows showed high reactivity using ELISA and HI tests against all the tested strains. These serological results were also observed in piglets tested at 4 weeks of age, while in Batch 3 only, the pigs sampled at 9–10 weeks of age showed positivity. The reactivity was against the two strains isolated in the farm: H1C2.4N2 (genotype T and genotype D) (Table 2; Fig. 7a) but seroconversion was not observed (data not shown).

The data collected on Farm#2, from the 6 sampled batches, showed a broad reactivity of the sows using ELISA and HI tests against all the tested strains (Fig. 7b). The maternal immunity, observed in samples collected from piglets at 4 weeks of age, decreased at 9–10 weeks of age. Seroconversion was observed in animals from Batch 3 against the strain H1C2.4N2-Gen35, isolated in the farm in 2023, which was detected in the same farm in Batches 3, 5, and 6 in the sampling period (Fig. 7).

### IAV-specific IFN-γ secreting cell response − 2022

**Fig. 8:**
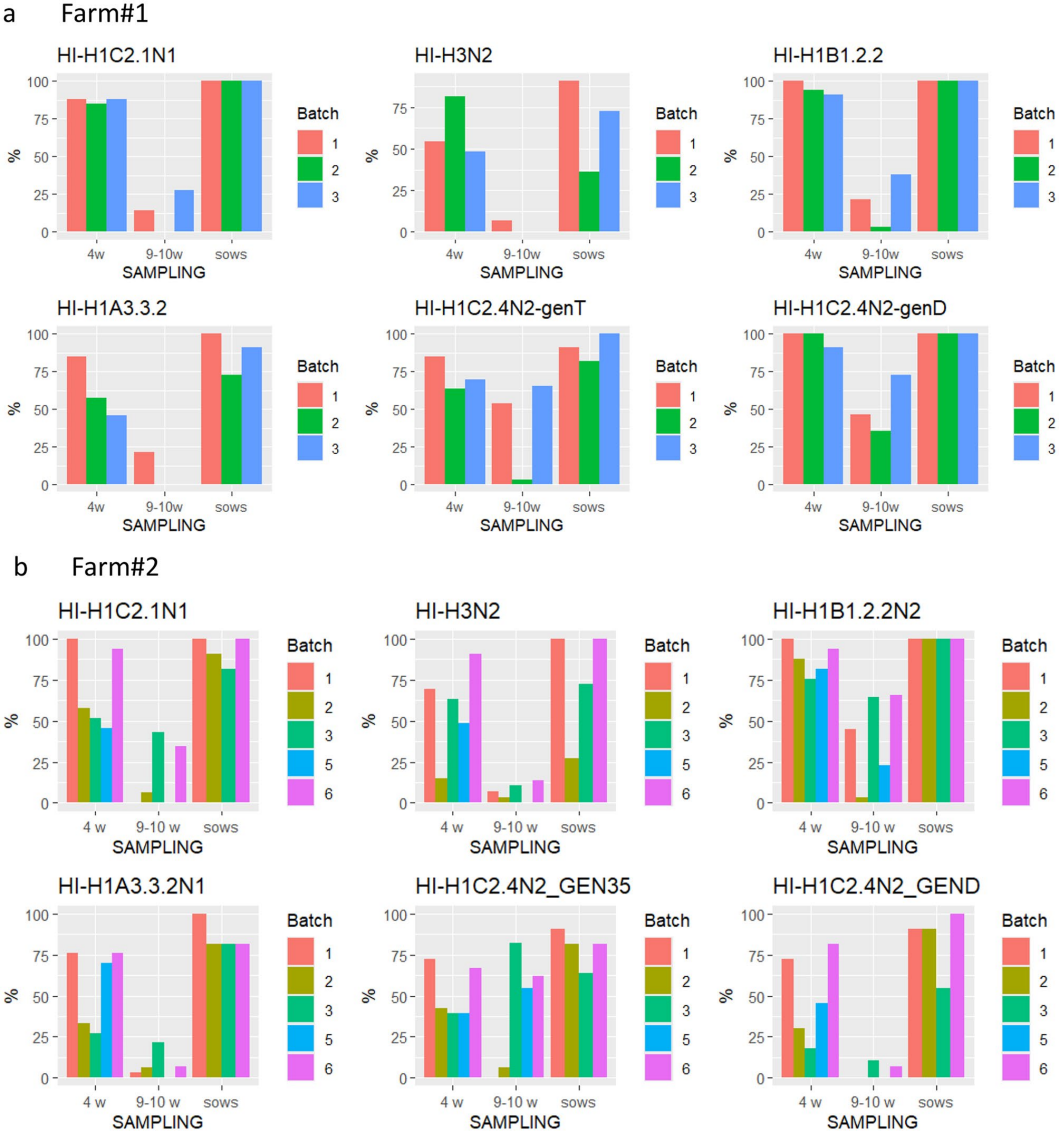
Percentage of positive animals in HI tests against the different viral strains in the different sampling groups (4 weeks, 9–10 weeks) and sows in Farm#1–2023 (**a**) and in Farm#2–2023 (**b**)

PBMCs from sows in both batches responded to stimulation with all strains (Fig. 8). The response in piglets was lower, but the trend was similar to the corresponding sows (Fig. 8). Moreover, the highest reactivity was recorded when testing the H1A3.3.2–2022 and the H3N2 strains, while the H1C strains induced a lower response, and the H1C2.4N2_2022 strain showed the lowest reactivity compared to the other strains tested (Wilcoxon test *p* < 0.05).

Data from piglets showed that the reactivity against the farm strains H1C2.4N2 2022 A/swine/Italy/49701–6/2022 and H1C2.1N1 2022 A/swine/Italy/49701–24/2022 was significantly lower (*p* < 0.05) than the responses against the H1A and H3 strains tested: H1A3.3.2N1 2022 A/swine/Italy/29548–31/2022, Routine H3N2 A/swine/Italy/311349/2013. No increase in response was observed at 9–10 weeks of age.

### IAV-S-specific IFN-γ secreting cell response − 2023

**Fig. 9:**
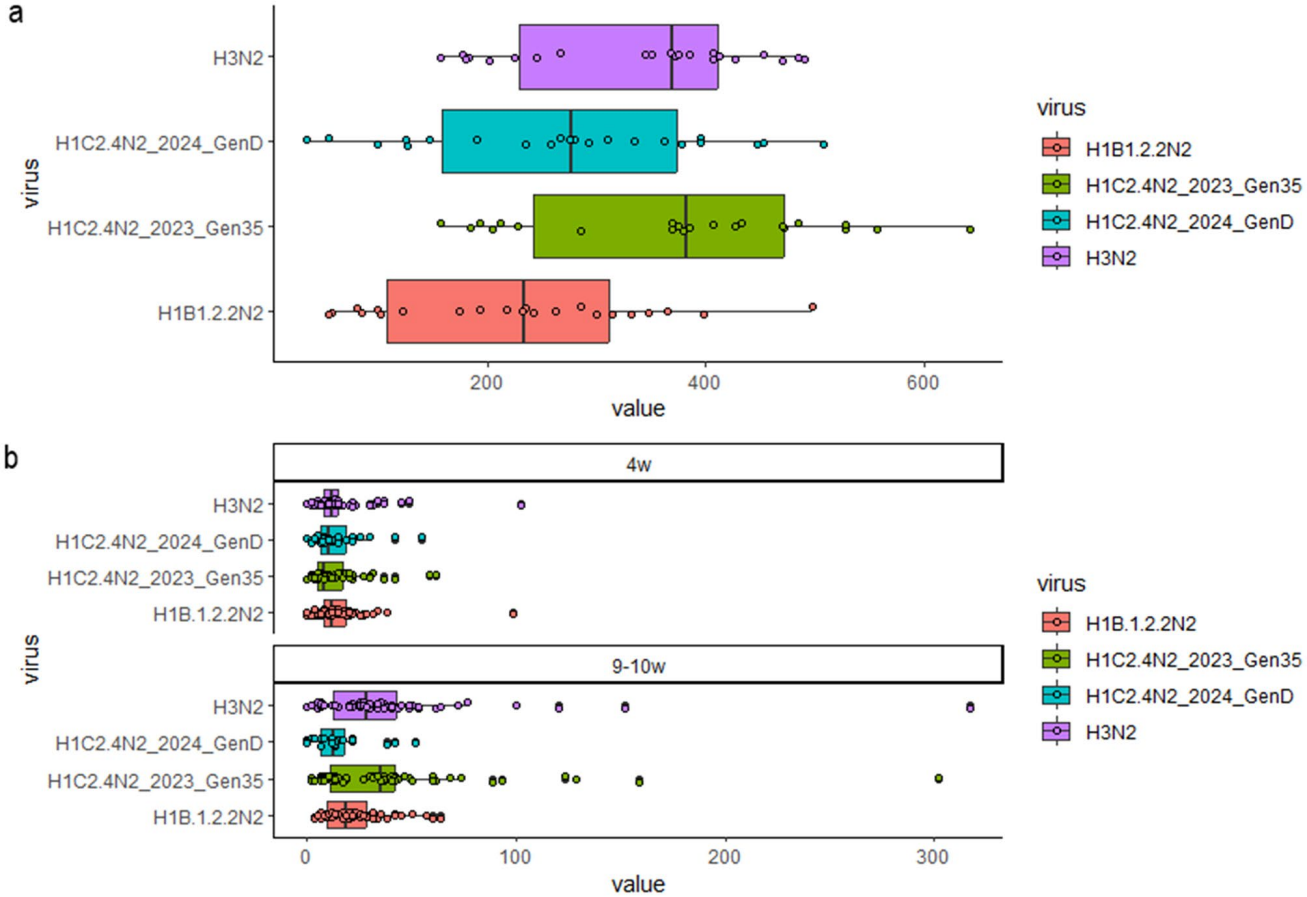
**a**) ELISpot results in sows. IFN-γ secreting cells/10^6^ PBMC for the two batches of Farm #2–2023 sows. Cells were stimulated with 0.5 MOI of four viruses. **b**) ELISpot results in piglets. IFN-γ secreting cells/10^6^ PBMC for the two batches of Farm #2–2023 piglets at the two sampling times: 4 weeks (4w) and 9–10 weeks (9-10w). Cells were stimulated with 2 MOI of four viruses: routine H1B1.2.2N2 A/swine/Italy/284922/2009, Routine H3N2 A/swine/Italy/311349/2013, and A/swine/Italy/299242-4/2023 H1C2.4N2_2023_Gen35 (from Farm #2) and H1C2.4N2_2024_GenD (from Farm #2)

Sow PBMCs responded to stimulation with all strains. A few differences among strains were detected, but none of the tested strains stimulated a higher response than the others (Fig. 9a).

Piglets showed reactivity against the strains used, and the response detected in PBMC at 9–10 weeks of age was higher than the response detected at 4 weeks (Wilcoxon test *p* < 0.05) with almost all the strains used (3 over 4) (Fig. 9b).

## Discussion

This study provided data on the circulation of IAV-S in two pig farms in Italy, collected through active sampling over 2 years and accounting for the immunological status of the sampled animals. One limitation of this longitudinal sampling is that it followed only one defined age group over time, while other age groups, such as fatteners, present on the farm at the same time were not sampled. Since cross-sectional studies have shown that different subtypes may circulate concurrently in distinct age groups [40], we cannot be sure that a strain detected at a later stage represented a genuine new introduction or whether the virus was already present in other age groups.

Nasal swabs were chosen as a reliable, cost-efficient, and convenient sampling method, and, even though they are highly sensitive to environmental contamination (e.g., dust) and do not reflect viral load in the lower respiratory tract [40], they can still be helpful in showing virus variability within the groups sampled. In fact, the longitudinal study conducted before vaccination implementation revealed a complex intra-farm epidemiological scenario, characterized by the co-circulation of multiple swine influenza lineages across both farms. In 2022, H1A3.3.2N1, H1B1.2.2N2, H1C2.1N1, H1C2.5N1, H1C2.4N2, and H1C2.2N2 were detected in the two farms. Initial screenings conducted one month before surveillance began identified one persistent strain on each farm, which was also detected throughout the monitoring period, indicating its continuous presence in the herds. Conversely, various IAV-S strains were also detected and the multiple detections observed in both farms confirm the data regarding the high genetic variability of the different IAV-S strains circulating in Italy [16]. In fact, we detected all the subtypes and all the H1 genetic clades circulating in the country (i.e., 1A3.3.2, 1B1.2.2, 1C2.1, 1C2.2, 1C2.4, 1C2.5), except for H3N2. The detected strains were reassortants of different origins, and the combination of internal genes and the neuraminidase gene differed.

As previously shown [12, 41, 42], it cannot be assumed that MDA will stop viral circulation; in fact, many piglets in one batch from each farm were detected as positive for viral presence in nasal swabs in the farrowing unit, without showing a detectable serological response by HI. MDA can, in fact, suppress or mask the piglets’ humoral immune response (as measured by ELISA or HI), resulting in seronegativity despite active infection. By 10 weeks of age, most animals were seropositive for NP antibodies. However, among unvaccinated animals, one case of seroconversion only was observed, on Farm #2, against the strain HC2.4N2 (isolated from the same farm), characterized by a double amino acid deletion (∆147,173) in the HA1 region. And again, the Farm#2 vaccinated piglets that showed seroconversion in 2023 were infected by H1C2.4N2 (Gen 35) strain, a ∆173 deleted strain. These strains (HC2.4N2) belonged to an emerging clade in Italy [16], and their effective infection in the herd could be due to the lack of passive immunity transmitted from the sows to their piglets [41]. However, because no control group was included in the study, we could only speculate over why, in the 2023 follow-up, we detected broad HI reactivity in sows against strains distant from the vaccine antigens [43]; even if this observation can’t be linked to a vaccination failure, the presence of H1C2.4 strains in both farms, before and after vaccination, suggests that this lineage could have a good fitness and is likely capable of evading immune defenses.

A longer monitoring period would be required to assess whether vaccination leads to durable changes in circulation patterns or only transient shifts in dominant genotypes, but the circulation of various lineages of the H1C2.4 subtypes (H1N1 and H1N2) highlights the importance of continuous surveillance and characterization of circulating strains within a given geographical area. These strains showed diversification, with deletions and mutations in the antigenic sites of the HA1 region that must be monitored in the future because they could lead to the emergence of new viral strains of influenza [44].

The farms in this study didn’t introduce new animals and had a complete farrow-to-finish cycle; therefore, the source of viral diversity must be investigated. Air transmission, personnel, and feed sources may be possible routes of introduction, but require further investigation. Some strains can persist at very low prevalence within a herd, making detection difficult [45]. Because virus sampling in this study focused mainly on suckling and nursery pigs, fatteners, which were not managed in strict all-in/all-out systems, may also have served as sources of these strains. As observed in other studies, despite the presence of maternal or vaccinal immunity, viral circulation in the groups remained active, and genetic diversity was maintained [46]. It is also important to highlight that the vaccination performed in piglets during the suckling period in this study protect them for a maximum of approximately 4 months. This leaves them susceptible to late infections during the fattening period, making them a possible source of viruses.

Similar studies have been conducted in different geographical areas (Denmark, Germany, Spain, the most critical United States, and Northern Ireland) [13, 47–49] and in different production systems in enzootically infected herds, showing a common pattern of viral persistence. These studies converged in showing that the weaning/nursery phase was the crucial point for virus circulation, with the highest virus elimination registered in the post-weaning/nursery period (4–12 weeks of age). Circulation was sustained by individuals clearing the virus for prolonged periods [47] and by recurrent reinfection events [45, 50, 51], even with almost identical strains [50]. Despite what has been observed in Ireland and Denmark, we did not detect any persistently infected animals. However, similar to Germany and Denmark [47, 52] the co-circulation of several virus variants was reported, and the infection dynamics involved different waves of several circulating strains. The common challenge was that viral circulation often occurred in the presence of MDA, which complicates vaccine protection and efficacy, and promotes the emergence of variants through antigenic drift [49].

Both sows and piglets mounted virus-specific T-cell responses, as shown by the detection of IFN-γ-secreting cells upon in vitro stimulation with the virus. This response indicates the activation of both CD4+ and/or CD8+ T cells, which are key players in cellular immunity against viral infections [43]. This activation was particularly evident in the group of vaccinated piglets born to vaccinated sows, which showed an increase in response at 9–10 weeks of age. The observed reactivity showed some strain-related differences but was broadly present because it was a multi-peptide stimulation and multi-subtype cross-reactivity was demonstrated.

Traditionally, booster vaccinations utilize the same vaccine formulation as the initial dose. However, studies on this subject [43, 53, 54] showed that administering booster doses with a different swine influenza virus could potentially broaden the antibody response, but, as previously demonstrated, vaccination doesn’t achieve sterile immunity [46, 49]. Interestingly, vaccination may significantly decrease nasal shedding of infectious virus and mitigate clinical disease and/or lung pathology. However, studies have shown that animals remain infectious even if vaccinated, with the possibility of virus transmission to other susceptible animals or humans, even when shedding levels are lower [46]. Even if it was not the aim of the study, evaluating the effectiveness of vaccination under field conditions cannot be meaningfully assessed because clinical and performance data were not considered. Our study was limited to detecting viral circulation in the two farms after vaccine introduction.

Since pathogenicity and zoonotic impact are difficult to predict, surveillance and epidemiology remain important tools for herd management and One-Health monitoring during the inter-pandemic phase. As evidenced in this study, the lack of reactivity of all recent isolates against hyperimmune sera produced by circulating human H1A3.3.2N1 strains suggests that the human population may be missing immunological protection from vaccine strains or from recent infections with the endemic H1A3.3.2N1 virus. Despite widespread vaccination efforts, swine influenza continues to pose a significant threat to both animal and public health, much like seasonal influenza in humans. The emergence of novel swine influenza viruses that can infect humans raises concerns about potential pandemics [7] and underscores the unpredictable nature of pandemic emergence and the importance of global surveillance [2, 55, 56]

## Electronic supplementary material

Below is the link to the electronic supplementary material.

Supplementary material 1

## Data Availability

The sequences of the strains have been deposited in GenBank under the Accession numbers: PV174958-PV175237, PV186487-PV186652, PP864066-PP864073. Raw serological and molecular data are available in Supplementary file S1.

## Declarations

### Dataset

The datasets are available from the corresponding author on reasonable request.
